# No oral health without a workforce? A public health perspective on the UK oral health workforce

**DOI:** 10.1038/s41415-026-9947-5

**Published:** 2026-11-13

**Authors:** Jennifer E. Gallagher, Najat Al-Katheri, Victoria Niven

**Affiliations:** King´s College London, Faculty of Dentistry Oral and Craniofacial Surgery, Centre for Host Microbiome Interactions (Dental Public Health), London, United Kingdom

## Abstract

The aim of this paper is to provide a public health perspective on achieving a sustainable oral health workforce in the UK. This involves taking a strategic perspective on need, demand and supply, drawing on contemporary sociological theory on professions and workforce governance.

Despite having more dental professionals than ever before and a diverse skill mix, people living in the UK are challenged to access to dental care, and disease levels are rising among adults. New models of professionalism and workforce governance are emerging, all of which are impacting what it means to be a health professional in a higher income country at this point in the 21^st^ century. Multiple pressures, including demographic, political, economic, technological, and environmental drivers, necessitate strategic transformation while also shaping professions.

A future sustainable workforce will require systems redesign, organisational and workforce development to align with population and professional need. A renewed focus on innovation embracing prevention, equity, humanity, professional engagement, and effective leadership will enable the dental workforce to be reshaped over time to serve current and future generations more effectively. There is indeed no oral health without a workforce but there is also no sustainable workforce without systemic reform.

**Supplementary Information:**

Zusatzmaterial online: Zu diesem Beitrag sind unter https://doi.org/10.1038/s41415-026-9947-5 für autorisierte Leser zusätzliche Dateien abrufbar.

## Key points

- The oral health workforce with seven professional groups is a vital resource, yet under pressure and struggling to meet population needs and demands.
- The rapidly changing context and the unique interplay of organisations and actors, and drivers for change make for a complex environment.
- A needs-led public health approach requires action from government, organisations and dental professionals to develop a sustainable workforce.

**Supplementary Information:**

Zusatzmaterial online: Zu diesem Beitrag sind unter https://doi.org/10.1038/s41415-026-9947-5 für autorisierte Leser zusätzliche Dateien abrufbar.

## Background

There is ‘no health without a workforce' declares the World Health Organization^1^ and ‘no health without oral health'.^2^

The aim of this paper is to provide a public health perspective on achieving a sustainable oral health workforce in the UK. This involves taking a strategic perspective on need, demand and supply, recognising the multiple drivers for change, underpinned by theory on the sociology of professions and contemporary workforce governance.

First, we will provide a brief overview of the size and shape of the current workforce considering ‘where are we now?'; second, analysis of the multiple drivers for change affecting the oral health workforce, i.e., ‘what is driving change?'; and third, propose future considerations in planning for a sustainable workforce, ‘what next?'.

## Where are we now?

Dentistry has evolved from within the shadows of medicine as a profession and over time diversified its skill mix such that the UK dental workforce, with some over 130,000 registrants, has seven groups of dental professionals and 13 listed specialties.^3^

The ‘dental team' has expanded to support a greater skill mix of dental professionals (dental therapists, dental hygienists, orthodontic therapists, clinical dental technicians, dental technicians), collectively known as dental care professionals (DCPs). All seven groups have been formally registered since 2008 (Table 1). The oral health workforce is, thus, highly professionalised and regulated.^4^

**Table 1:** UK registered dental workforce

**Registration type**	**Dec-08**	**Dec-24**	**Dec-25**	**Mar-26**	**Workforce: population ratio Nov-25**
Dentist	32,281	46,309	47,901	47,332	1:1,468
Dental care professionals	56,880	79,140	83,593	84,714	1:820
Dental nurse	42,599	64,956	68,312	69,368	1:1,002
Dental technician	7,460	5,016	4,966	5,000	1:13,897
Dental hygienist	5,160	10,460	11,291	11,322	1:6,137
Dental therapist	1,164	7,313	8,657	8,715	1:7,974
Clinical dental technician	121	439	459	463	1:150,080
Orthodontic therapist	16	1,154	1,301	1,316	1:52,802
**All**	**91,548**	**125,449**	**131,494**	**132,046**	**1:526**
Source: GDC, 2009, 2024, 2025, 2026; ONS, 2025 Note: One registrant may be registered under more than one professional title; therefore, the sum of DCP registrations may exceed the total number of individual DCP registrants shown in the table above					

**Fig. 1:**
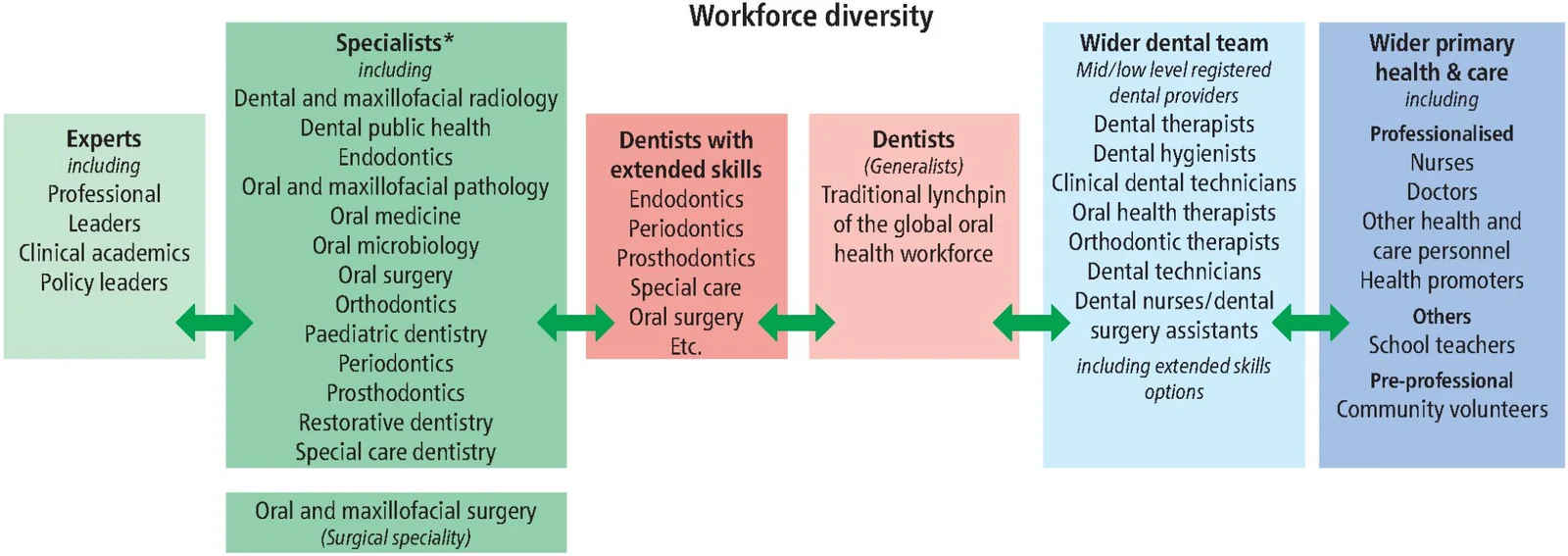
Unique UK oral health workforce: diversification. Adapted from Gallagher J. E., from a global workforce model presented at WHO Workforce Side Event, Bangkok, November 2024. (*The 13 dental specialties recognised by the General Dental Council are shown alongside Oral and Maxillofacial Surgery, a surgical specialty in the UK)

With one dentist per 1,468 people, one DCP per 820 and, thus, one dental professional per 526 people, the UK has a sizeable and potentially influential workforce, albeit not as large as some other European countries.^5^ Furthermore, we can draw on a wide range of other health and care professionals and community players to support oral health through health promotion (Fig. 1).

The number of registrants, however, does not represent their contribution to the health systems. The recent General Dental Council (GDC) workforce survey^6^ reported that engagement with the NHS, mixed, and private care varies, as does the level of part-time working, across professional groups. Interestingly, a higher proportion of dentists' report having a commitment to the NHS and full-time working than providers such as dental therapists and dental hygienists (Table 2).

**Table 2:** Dental professionals' GDC-working pattern data

**Professionals†**	**Registrants, respondents (%)**	**Main sector of work‡**	**Working hours (full-time versus part -time)§**	**Key additional highlights**
Dentists	45,428 registrants; 30,066 respondents (66%)	NHS 42% Mixed 22% Private 34%	FT 54% PT 44%	59% self-employed/locum workers, and 16% owners or part-owners of the business 38% work in more than one location
Dental nurses	65,862 registrants; 46,474 respondents (71%)	NHS 28% Mixed 38% Private 33%	FT 55% PT 45%	88% employed. 81% work in one setting. 46% work fully clinical; 24% work a mix of clinical and non-clinical work
Dental hygienists	11,089 registrants; 7,795 respondents (70%)	NHS 12% Mixed 24% Private 63%	FT 36% PT 63%	68% self-employed/locum workers 59% work in more than one location 30% primarily practise as dental therapists
Dental therapists	8,485 registrants; 5,384 respondents (63%)	NHS 20% Mixed 33% Private 45%	FT 44% PT 55%	65% self-employed/locum workers 56% work in more than one location 37% primarily practise as dental hygienists
Orthodontic therapists	1,258 registrants; 965 respondents (77%)	NHS 46% Mixed 43% Private 11%	FT 55% PT 45%	75% are employed 64% work in a specialist dental practice 37% work in more than one location 10% primarily practise as dental therapists
Dental technicians	4,899 registrants; 3,051 respondents (62%)	NHS 21% Mixed 35% Private 40%	FT 80% PT 18%	61% are employed, and 22% are owners or part own the business 67% work in a lab setting, 10% work in a dental hospital and 8% work in general dental practice
Clinical dental technicians	440 registrants; 299 respondents (68%)	NHS 3% Mixed 20% Private 73%	FT 67% PT 31%	41% are owners or part-owners of the business, and 31% are self-employed/locum workers 41% work in more than one location 45% work in a lab setting, and 30% work in general dental practice
FT, full-time; GDC, General Dental Council; NHS, National Health Service; PT, part-time. Data source: GDC https://www.gdc-uk.org/about-us/our-organisation/reports/working-patterns-data. Dentist data published in April 2025, and dental care professional data published in October 2025. ^†^A dental professional may be registered under multiple titles with the GDC, but the working patterns question specifically asks for their primary field of practice. ^‡^‘Fully NHS' and ‘predominantly NHS (≥75%)' groups were combined as ‘NHS'; and ‘fully private' and ‘predominantly private (≥75%)' groups were combined as ‘private', for the ‘main sector of work'. ^§^Percentages may not sum to 100% because responses of ‘I don't know' and ‘prefer not to say' are not reported.				

Comparing NHS data from the four nations suggests that most dentists still deliver some NHS care, albeit falling. In March 2025,^3^ the share of dentists with a registered address who had an NHS primary care relationship was lowest in England (67.3%),^7^ and highest in Wales (80%),^8^ with Scotland (77%),^9^ and Northern Ireland (70.7%),^10^ in between, indicating differences between the four systems and contexts. Within countries, the demand for dental care is higher than supply and this is most evident in rural and coastal areas.^11^

The evolution of dental specialties is in response to population need, demand and innovative opportunities.^12^ About 10% of dentists have listed specialist titles and this level of specialisation has remained stable for some time.^3^ Oral and maxillofacial surgery continues to play an important role in trauma and head and neck surgical care. GDC specialties are largely based on highly regulated training and within the NHS delivered according to care pathways which may also involve dentists with enhanced skills.^13^ Not all specialties are hospital-based; many, such as orthodontics, are delivered in primary care settings, across public and private sectors.

It is interesting to reflect on why people embark on a professional degree in dentistry. Research suggests that students continue to apply to study dentistry in the UK for a myriad of reasons including: providing a vocational, science-based career, with status and security; career opportunities; social interactions; personal skills and care; with individual, social, community and societal factors influencing these perceptions.^14^^,^^15^ These perceptions likely influence career expectations, with implications for long-term career satisfaction. Dental careers do not necessarily turn out as envisaged.^16^

Dental nurses and dental hygiene therapists report similar motivations, including working with people, job security, contributing to healthcare, and opportunities for professional development within dentistry.^17^^,^^18^ These shared drivers reflect altruistic and pragmatic motives.^19^

Workforce retention is increasingly difficult. Evidence, even pre-pandemic, suggested that the health and wellbeing of dentists^20^^,^^21^^,^^22^ and DCPS^23^^,^^24^ were challenged by working in systems and organisations where they raised concerns about their physical and psychological health,^20^^,^^21^^,^^22^^,^^23^^,^^24^ and most notably the lack of control.^23^^,^^24^^,^^25^ It is clear that personal resilience alone will not suffice, and improving the culture affecting front-line staff, together with career development^26^ will be critical for enabling staff to find a fulfilling career.

Clinical training numbers are capped nationally, and there is a new move to increase the dentist workforce in England including boosting opportunities for international dentists to gain qualifications to enable registration with the GDC.^27^ However, additional workforce numbers will need to be funded to deliver care on the front line,^5^ and there are geopolitical and ethical considerations associated with workforce movement from other countries.^28^

Thus, the oral health workforce, public, patients and governments are wrestling with complex interplay between need, demand and supply.

## What is driving change?

**Fig. 2:**
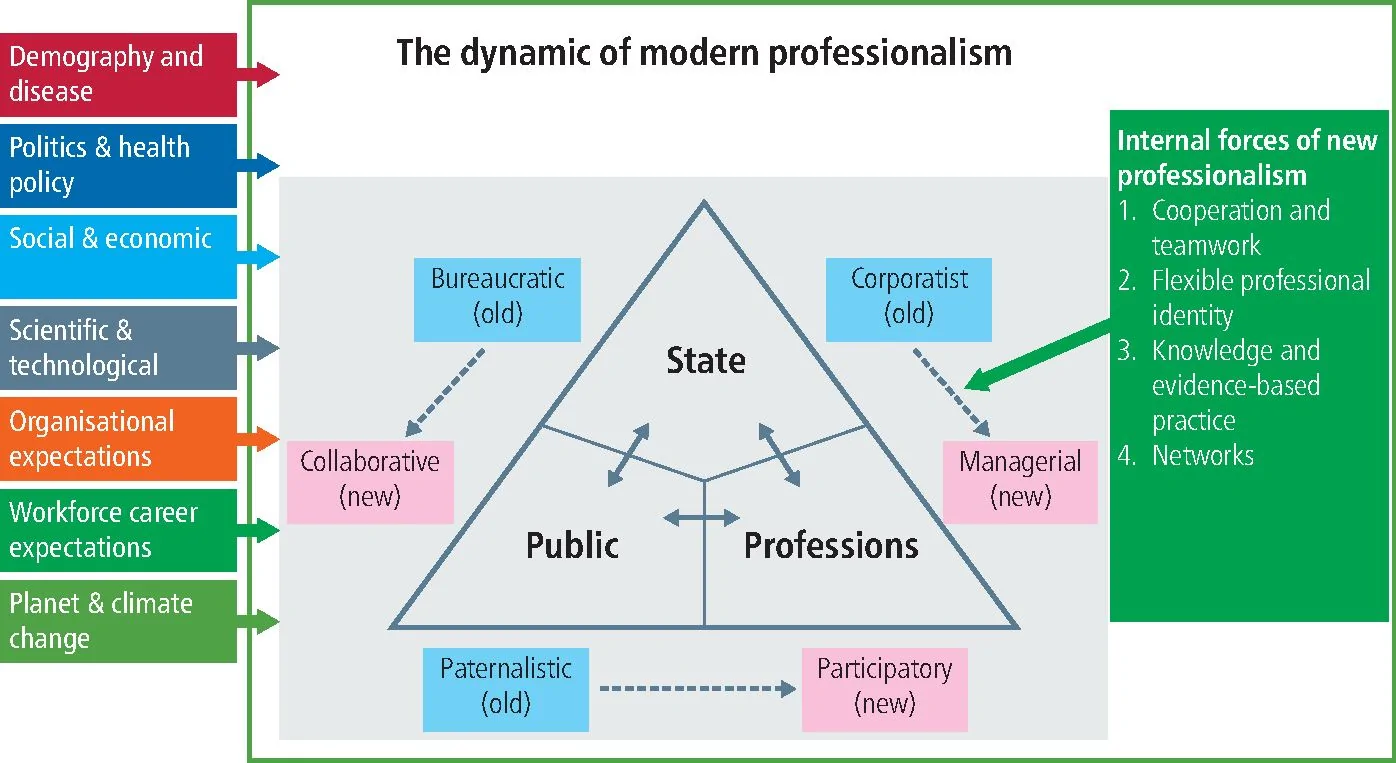
Drivers for Change on Dynamic Triangle of Professionalism (old and new) (adapted from Kuhlmann's framework of Modernising Health Care. Interaction between the state, professions, and public, illustrating internal and external forces/drivers shaping modern professionalism and the shift from corporatist-paternalistic relations to collaborative-participatory governance)

Pressures appear to have exacerbated since the COVID-19 pandemic.^29^ And it is important to recognise that the challenges faced by dentistry are not unique to our nations or our branch of healthcare.^30^ To deepen our understanding it is helpful to draw on the sociology of professions and workforce governance and to explore the multiple drivers for change and their implications. The interplay between state, profession and public is vital to understand, particularly as professions are, without realising it, negotiating a transition from traditional to new professionalism (Fig. 2).

## Reframing professionalism: from traditional to new models

### Traditional professionalism

Professions became established in the UK during the 19^th^ century with the rise of the middle classes, and traditionally embarked on what sociologists call they ‘professional projects',^31^^,^^32^ driven by the need and opportunity to establish a professional group for a shared purpose and thus to achieve ‘status in the social and economic orders', i.e., ‘power and money'.^31^^,^^32^ This involved gaining a monopoly over a particular ‘jurisdiction' (area of work),^33^ securing legal closure through credentialing and control of entry, and crafting opportunities to educate and train the next generation; thus, ‘producing the producers' and sustaining the profession.^31^^,^^32^ In dentistry, however, only dentists fully achieved professional status in this traditional sense.^31^ This sociological perspective doesn't perhaps take account of the humanitarian drivers of healthcare professionals but it does help to understand something of the dynamics of professions.

### New professionalism and governance

Kuhlman^34^ offers an interpretation of recent developments in professions; developments that many of us have lived through but not fully understood. Her insights to the wider health workforce suggest that new forms of governance, along with new forms of professionalism and professional identity, are emerging. They include teamwork, networking, and a greater emphasis on guideline-driven care, all within an environment of marketisation, commercialisation, managerialism, corporatisation, and cost containment (online Supplementary Table 1).^34^ The triad of relationships is transitioning from a bureaucratic, paternalistic and corporatist model, to a newer model where professionalism is becoming more collaborative, participatory and managerial (Fig. 2).

Our expanded skill mix is part of the new professionalism, facilitated by government, regulatory and professional collaboration, and followed by formalisation of an agreed ‘core' Scope of Practice,^35^ which continues to be revised.^36^^,^^37^ Dentists now share their ‘jurisdiction', i.e., scope of practice with six other members of the dental team. It has been recognised for some time that all dental professionals can gain additional skills;^35^^,^^36^ thus, having the potential to expand their jurisdiction and enhance careers.^37^ Interestingly, the recent 2025 Scope of Practice, has moved away from listing specific tasks for each group towards providing more flexibility for dental professionals to develop their base skills from the launchpad of their respective Safe Practitioner Framework over time, requiring all to be ‘trained, competent, and indemnified, to provide safely ‘any additional tasks' they undertake.^37^ Skills, competencies and tasks of dental professionals overlap and, in sociological terms, this is called having a ‘shared jurisdiction'.^33^^,^^36^^,^^37^ This guidance suggests less defined and increasingly ‘porous boundaries' between professional groups, presenting challenges for day-to-day working, sharing patient care, professional status and remuneration as well as significant opportunities for patient care.^37^

### Models of care delivery

Historically, dental practices have largely operated in isolation from one another, with pathways limited to referring on to specialists in a vertical manner; however, new relationships are emerging. First, the move away from small independently owned practices towards large groups or corporate bodies (practices with special legal status as an organisation with limited or defined liabilities) means more limited opportunity for dental professionals to establish their own practice unless they have private financial means and greater likelihood of working in managed teams. Second, care pathways from primary to secondary care are now system-based rather than reliant on individual referrals, having dentists with enhanced skills in areas such as oral surgery, endodontics and special care dentistry, often termed ‘Level 2 dentists', providing care between generalist and specialist practice.^13^ Furthermore, dentist-led networks of NHS care through associations and local ‘managed care' networks have taken clinicians out of their silos to connect across health systems from generalists through special interests, to specialists, meeting with commissioners and dental public health consultants to shape coordinated care across the system, achieving benefits for all, and reducing potentially isolation.

Some professionals have adapted well to these new models of professionalism, leading the development of guidelines, building networks, embracing teamwork and patient-centred care (Fig. 2). However, others on the frontline experience growing pressure from managerialism and corporatisation. Having less control and autonomy, may be prompting some to consider leaving the profession or shifting toward the more aesthetic or private sectors of dentistry.

### Key drivers of change

Multiple drivers of change require consideration (Fig. 2) to understand current workforce-related challenges and inform future deliberations between the triad of professions, public and government.

### Demography and disease

Central to future workforce considerations is the recognition that we are serving a population that is both expanding and ageing, with diverse needs and expectations. People are living longer and retaining some, or all, of their natural teeth.^4^ However, marked inequalities in oral health remain,^38^ and the recent findings of the Adult Oral Health Survey 2023 for England^39^ make stark reading, highlighting the volume of untreated disease across all sections of society but particularly among lower income groups.

Future dental care models should thus extend beyond merely one-off courses of treatment, to consider health across the life-course, underpinned by evidence-based prevention^40^ and include planning for older age.^4^ Furthermore, self-care is clearly ‘less than optimal'.^39^ Much of this not surprising given social and commercial determinants of health. This calls for an increased orientation towards prevention to manage the large burden of disease effectively.

### Scientific and technological change

Dentistry is rapidly advancing through the emergence of digital technologies,^41^ biotechnology,^42^ tele-dentistry,^43^ and artificial intelligence (AI).^44^ The challenge is how can we harness the range of emerging technologies to make better use of our professional time and improve care from record keeping to prevention and minimise costs. All of this will require health professionals to keep refreshing their skills and expertise, with implications for lifelong continuing professional development.

### Social and economic change

Society has always had an interesting relationship with health professions and health systems. Citizen consumers are better informed and more demanding of choices from clinicians and the government, and ready to complain when expectations are not met. Currently demand for care exceeds supply.^39^ Societal aesthetic expectations of oral ‘health', perhaps fuelled by social media, are driving the dental tourism industry and creating challenges for the NHS.^45^ The social value of dentistry is apparent for some but not all elements of dentistry – perhaps more so for urgent care and the aesthetic side of dentistry rather than health. How in future can we help patients and the public to value timely reparative dentistry and preventive self-care to maintain oral health effectively into older age?

Financial pressures abound, yet every recent workforce report in Europe suggests that greater financial resources are required to retain a quality health workforce within the healthcare systems generally,^30^ whether in the state or private sector. As a society we must face up to this if we want healthcare systems fit for a higher income country. And an expanded workforce will require additional educators and trainers.

### Politics and health policy

The driving pressures of neoliberalism^34^^,^^46^ have resulted in major changes in government and welfare-state policy, and the ever-increasing governance of the public sector and organisational frontline management with a view to managing NHS costs and improving performance. Successive governments have endeavoured to tackle problems through organisational systems reform, which, ironically, interferes with facilitating change on the front line. The effect of multiple reorganisations is taking its toll, particularly in England. Our specialty of dental public health knows only too well the pain of reapplying for jobs in the new system.^47^ Furthermore, there is the recognition that ‘the NHS cannot performance-manage its way out of exhaustion' and without addressing deeper issues relating to ‘morale' and ‘chronic overload', ‘structural reform will not hold'.^34^

Healthcare reform has prioritised finance as the primary management tool, often neglecting the central role of the health workforce.^48^^,^^49^ National bodies should ideally ‘lead differently, through trust, learning and shared purpose' for healthcare recognising the commitment of staff.^46^ And government must go further in tackling the wider psychosocial and commercial determinants of health.

More optimistically, oral health sits higher than ever in government policies in response to unmet public demand.^50^^,^^51^ Stronger engagement of dental public health in commissioning and system design will be essential for the future to ensure a focus on addressing need.^47^^,^^52^

### Organisational expectations (providers)

Given that the business of health care drives action and particularly in a contemporary neoliberalist culture,^34^ the effect is increasingly evident within large contemporary dental organisations, where staff are heavily performance managed.^25^ New governance requires an engaged workforce and collaboration across providers rather than merely competition. Can providers be inspired to work on innovative new models of care embracing team working in a thriving culture; and, thus, provide exemplars of contemporary care for others?

### Individual workforce expectations

Expectations of work/life balance are key to the future,^16^ and yet we have a multigenerational workforce with differing views of professional careers and jobs. All organisational expectations will need to reshape to harness ‘Generation Zʹ^53^ among, whom a staggering 86% see ‘purpose' as pivotal to their overall wellbeing and job satisfaction. There is much purpose to be found within dentistry from developing effective team working to delivering sustainable dentistry and proactively providing care to those who most need it. Students select their career as an ‘altruistic' vocation, perceiving that it provides autonomy over work/life balance; thus, retention may depend on meeting these expectations.^54^ We need to explore how to enhance career development of our expensively educated and trained dental professionals to facilitate their engagement, while recognising that many are unlikely to work full time. This has implications for education and training organisations and the public purse.

### Planet and climate change

Human health is inseparable from the health of the planet, one of the most important issues of our time. Dentistry has an important role to play;^55^ there is much to be done in addressing key sources of emission including travel, procurement and waste. Embracing health promotion is fundamental to sustainable dental care, as well planetary care, and we have a strong contemporary evidence base in ‘delivering better oral health' to assist further orientation towards prevention.^40^ Furthermore, patients and the public need support to recognise their responsibility for effective self-care in support of their health and the health of the planet.

## What next – how do we plan for a sustainable workforce in future?

Oral health is a human right.^56^ What value does society place on our workforce and the care they can deliver? Perhaps we as dental professionals need to encourage philosophical and evidence-informed debate on the future of oral and dental healthcare and do so on the concept of ‘health', not merely ‘aesthetics' or ‘business' priorities. The oral health workforce comprises very able human beings who have other dimensions to life which need to be combined with professional life. And if they can't do so in healthcare, they are smart enough, or able enough, to ‘move on'.

First, governments, policy makers and commissioners have a role to deepen their knowledge and understanding of oral and dental healthcare, finding the determination to make decisions that are of longer-term gain rather than short-term political expediency. The Global Strategy and Action Plan for Oral Health 2030 aligned with universal health coverage and non-communicable disease agendas, provides a helpful template for action.^56^ Strategic Objective 3 challenges member states to develop innovative workforce models. This involves moving beyond traditional, dentist-centred training towards competency-based, team-oriented education – we are already there. Closely allied to this is Strategic Objective 4, which aims to deliver new models of healthcare to support the achievement of universal health coverage for essential oral healthcare. Needs-led planning has long been advocated to support change.^57^ As a higher-income country, we are well placed to harness our professionalised workforce and to develop and test new models of care where health professionals can regularly work to the top of their competence and extend their skills over time. We have begun this journey and although dentistry is expensive to deliver, many of the interventions to support oral health are inexpensive, and vitally important for the future health of our nation.^38^ Learning from other nations, and finding ways of doing so sustainably (financially, environmentally and regarding workforce retention), will also be important. There is not a one fix solution but rather a process of change which values health professionals' contributions and shapes care in different contexts, underpinned by research.

Second, we, as dental professionals, can reflect and perhaps better understand the changes that are occurring and the importance of our roles in meeting the needs of our population, addressing inequalities in oral health and access to care, and caring for our planet. Leadership for change will be important, advocating to liberate dental professionals to bring their creativity into dentistry, facilitate the delivery of high quality oral and dental care in line with evidence and training across practice and community settings.^58^^,^^59^ This requires an engaged motivated workforce embracing high quality prevention-led, integrated models of care which address population need;^56^^,^^57^ and to radically push the boundaries of sustainable dentistry. Ongoing professional development will be required across the course of a dental career to build knowledge and skills and support motivation. While important for all, dental nurses are well placed to extend their knowledge and skills in health promotion.^60^ Together, this should enable dental professionals to take delight in delivering quality care and remain energised in their professional careers. Change is necessary; and it is worth remembering that professions are dynamic and notoriously good at reinventing themselves, when required.^31^

Third, organisations play an increasingly key role in this process of change. We would benefit from a shift in organisational culture to put humanity at the heart of workforce conversations, build trust, learning and shared purpose, restoring dignity, caring for staff and facilitating their development over time. Engaging the motivation, curiosity and creativity of younger generations of dental professionals in shaping care will be essential to build and retain an energised workforce. The business of dentistry, whether publicly or privately funded must embrace prevention and involve greater use of skill mix and the development of extended skills. Organisational initiatives to test and evaluate new models of care could provide helpful examples of good practice on the next phase of our professional journey.

## Conclusion

The UK possesses a highly professionalised oral health workforce; however, sustainability depends not on numbers alone but on system design, governance, morale, and alignment with population need. Multiple pressures, including demographic, political, economic, technological, and environmental drivers, necessitate strategic transformation while also shaping professions. A renewed focus on innovation embracing prevention, equity, humanity, professional engagement, and effective leadership, taking a public health perspective, will enable the dental workforce to be reshaped over time to serve current and future generations more effectively. There is indeed no oral health without a workforce but there is also no sustainable workforce without systemic reform.

## Supplementary Information

Diversity of professionalism between exclusion and social inclusion (PDF 173KB)
