# Supplementary material for: No oral health without a workforce? A public health perspective on the UK oral health workforce
Source: Br Dent J. 2026 Nov 13;241(9):667–73. doi: 10.1038/s41415-026-9947-5 (PMC13768168; doi:10.1038/s41415-026-9947-5)
Supplement: Supplementary file 1 [file 41415_2026_9947_MOESM1_ESM.pdf]

# Online Supplementary Table 1: Diversity of professionalism between exclusion and social inclusion

| Exclusionary patterns of professionalism | More inclusive patterns of professionalism | Examples from UK dentistry |
|---|---|---|
| Hierarchical, bureaucratic patterns of self-regulation and self-administration | Network governance, more active participation in the self-regulatory bodies | Dental profession moved from self-governance to embrace dental care professionals on the council and then multi-actor governance with lay leadership of the General Dental Council (GDC).<br>*GDC reform in 2009 replaced the elected dentist-only structure with an appointed Council of equal lay and registrant members[2]. |
| Striving for market closure | Cooperation | The traditional dentist's monopoly over treatment with delegated care has evolved into a shared-care model involving Dental Care Professionals (DCPs), and later to 'direct access', giving greater autonomy to some of the DCPs in an expanded skill mix model[3,4,5,6].<br>The latter included 'direct access' to dental care professionals such as clinical dental technicians, dental hygienists and dental therapists. |
| 'Tribalism' and occupational closure | Networking and more permeable occupational boundaries | The GDC's Scope of Practice[3] formally recognises additional skills of DCPs, breaking rigid occupational boundaries. Furthermore, the recent guidance on the scope of practice focuses on being competent, trained, and indemnified[6].<br>Regulations such as 'Standards for the Dental Team'[7], Guidance on Scope of Practice[6], and NHS guidance 'Building Better Dental Teams'[8] emphasise teamwork, appropriate delegation, and shared responsibility within dental care delivery. |
| Claims for 'autonomy' and self-determined decision making | Inclusion of managerialism, standardisation of care, EBM | Clinical decisions remain professional but are guided by measurable frameworks and evidence-based standards, such as NICE guidelines[9], Delivering better oral health toolkit for prevention[10], SDCEP publish guidance for oral healthcare[11], NHS contract Performance Indicators, Clinical audits. |
| Quality of care based on individual qualification | Quality of care based on formalised procedures and performance indicators | Quality is measured through Care Quality Commission (CQC), Healthcare Improvement Scotland (HIS), Healthcare Inspectorate Wales (HIW), and The Regulation and Quality Improvement Authority (RQIA) assessment framework[12], clinical audit, and patient feedback rather than relying only on clinician's reputation. |

| | | | |
|---|---|---|---|
| Identity construction based on ‘belonging’ to a professional community | ⇄ | More contextualised and permeable identity constructions | Team-based professional identity through ‘Standards for the Dental Team’; Published scope of practice from 2009 and becoming more porous in 2025 [3,4,6,7,13].<br>Dental team members may be registered as more than one type of health professional with the GDC [14]. |
| Gendered division of the health workforce | ⇄ | Changing gender relations within professions | Dentistry in general is now gender-balanced (≈52% female dentists), challenge traditional male–female hierarchies within dental teams [14].<br>Dental nursing which is predominantly female (~98%) has gained statutory registration since 2008 and formal career pathways; thus, increasing the number of female registrants with the GDC.<br>Across the specialties: females predominate in Paediatric, Special Care, and Orthodontics. Males predominate Oral Surgery, Endodontics and Restorative Dentistry [14]. |
| Expert–lay divide | ⇄ | Improved information, inclusion of users in regulatory bodies | Patients are regarded as informed users and citizens, rather than passive recipients.<br>Patient-centred care and shared decision-making are supported by transparent public information on services and clear complaints procedures. Public also have access to evidence-based oral health information (e.g., NHS.UK, Delivering Better Oral Health toolkit) to help patients understand prevention, treatment options, and costs.<br>Under the GDC ‘Standards for the Dental Team’, treatment options, costs, and consent must be discussed openly and documented to ensure patients are fully informed and actively participate in decision making [7].<br>Lay members sit on professional and health service boards, representing public interests [15]. |
| Professionalism restricted to the medical and other high-status occupational groups | ⇄ | Health occupations refer to professionalism to upgrade occupational status | All DCPs are highly professionalised; statutorily registered with the GDC, adhering to ethical codes, and continuing professional development to enhance their occupational status and argue for greater recognition, autonomy, and career progression within the dental team [7]. |

## Source References:


1. Kuhlmann E. Modernising Health Care. Reinventing professions, the state and the public. Bristol: Policy Press; 2006.
2. Stephen H. The GDC under the microscope. Faculty Dental Journal. 2016;7(3):98-102.
3. GDC. Scope of practice London: General Dental Council; 2009 [Available from: https://heeoe.hee.nhs.uk/sites/default/files/docustore/1279809682_ywJv_scope_of_practice_april_2009.pdf.
4. GDC. Scope of Practice London: General Dental Council.; 2013 [Available from: https://www.gdc-uk.org/docs/default-source/scope-of-practice/scope-of-practice.pdf.
5. GDC. Direct access London: General Dental Council; 2019 [Available from: https://www.gdc-uk.org/standards-guidance/standards-and-guidance/direct-access.
6. GDC. Guidance on Scope of Practice London: General Dental Council; 2025 [Available from: https://www.gdc-uk.org/docs/default-source/information-standards-and-guidance/scope-of-practice/scope-of-practice-guidance-2025-(1).pdf?sfvrsn=18b7c40e_1.
7. GDC. Standards for dental professionals London: General Dental Council; 2019 [Available from: https://www.gdc-uk.org/standards-guidance/standards-and-guidance/standards-for-the-dental-team.
8. NHS England. Building Dental Teams: Supporting the Use of Skill Mix in NHS General Dental Practice – Long Guidance. London: NHS England; 2022.
9. NICE. NICE guidance: National Institute for Health and Care Excellence; 2025 [Available from: https://www.nice.org.uk/guidance.
10. DHSC. Delivering better oral health: an evidence-based toolkit for prevention: Department of Health and Social Care & NHS England; 2014 [updated 10 September 2025. Available from: https://www.gov.uk/government/publications/delivering-better-oral-health-an-evidence-based-toolkit-for-prevention.
11. SDCEP. Published guidance for oral healthcare Scotland: Scottish Dental Clinical Effectiveness Programme & NHS Education for Scotland; 2025 [Available from: https://www.sdcep.org.uk/published-guidance/.
12. CQC. Memorandum of understanding – systems regulators for the four nations: Care Quality Commission; 2024 [updated 30 May 2024. Available from: https://www.cqc.org.uk/about-us/our-partnerships/joint-working-agreements/memorandum-understanding-4-nations.
13. Williams W FE, Edwards N,. Bold action or slow decay? The state of NHS dentistry and future policy actions. Policy briefing 2023.
14. GDC. Annual registration reports. London: General Dental Council; 2025.
15. GDC. Council members London: General Dental Council; 2021 [Available from: https://www.gdc-uk.org/about-us/who-we-are/the-gdc-council/council-members.
